# Rheumatism and Gout

**Published:** 1901-05-18

**Authors:** 


### RHEUMATISM AND GOUT.

(*Continued from page* 49.)

**Hæmophilia.**—C. Royds Jones[23] mentions a successful treatment with liquor thyroidei administered for six weeks, and refers to another case recorded by Delace. Gelatine has been given for the same purpose both internally and by subcutaneous injection. Schwabe[24] treated a case of hœmaturia with 2 per cent. of gelatine dissolved in 7 drachms of saline solution followed by a daily draught of a pint of 10 per cent. infusion of gelatine. The very rare cases of bony enlargement about the joints in hœmophilia were discussed at the Clinical Society. Percy Kidd[25] showed a patient in whom the Roentgen rays proved that bony outgrowths existed, thus negativing the theory of mere tubercular destruction, whether the condition had been started by hæmorrhages into the joint originally or otherwise.

**Purpura.**—Nehvkorn[26] records a hæmorrhagic case in a male, aged 26, which terminated fatally in four days with bleeding from the nose, mouth, bowels, kidneys, and into all the viscera. There was endocarditis, and the general appearances of an infectious disease. Spaight[27] mentions a child of 9 who while in apparently perfect health became covered with purpuric spots and during the next three days bled profusely from the orifices but recovered as promptly.

**Gout.**—A. Strumpel[28] holds that gout is much more common in Germany than is supposed. In diagnosis he refers to the very frequent affection of the toe, and the preference for the lower limbs and distal joints, the greater frequency and shorter duration of acute attacks, the suddenness and severity of the pain, and the fewness of the joints attacked at once. There are also cases of incomplete or concealed gout, where tophi, ankylosis of the little finger and irregular distortion of the fingers, contracted kidney and eczema may help in the diagnosis.

Beverley Robinson,[29] in speaking of the irregular manifestations of gout and of individual peculiarities as to diet took occasion to claim many cases of appendicitis as gouty. Abbe replied that if gouty peritonitis existed, why did it always occur on the right side of the abdomen? Moreover, in the apparently normal appendices which he had removed, microscopic examination always showed chronic catarrhal hypertrophy of the mucosa aud submucosa with strictures. J. D. Bryant added that pathological changes were found post-mortem in 60 per cent. of persons who died from various diseases. It is clear that these cannot be all due to gout; moreover gout and appendicitis are prevalent at different periods of life.

**Myosites Ossificans.**—Some 79 cases have been recorded. Rolleston[30] remarks that there is a local form where ossification takes place in a single muscle from a neighbouring inflammation. In the progressive form of the disease one muscle after another becomes inflamed, as in rheumatic myositis, but afterwards an area in each becomes calcified, or occasionally suppurates. Fixation of an increasing number of muscles ensues, and the patient becomes helpless and dies of some intercurrent disease. It arises from an interstitial myositis and must be distinguished from multiple exostoses, osteitis deformans, some cases of tabetic arthropathy, and rhizomelic spondylosis or anchylosed spine.

**Formation of Uric Acid.**—Halliburton[31] gave a summary of certain recent investigations, commencing with the work of Mares and Horbaczewski, who showed that much uric acid arises from a liberation of nuclein in leucocytosis which attends digestion, and that the spleen can oxidise alloxuric elements of nuclein into this acid. Furthermore, it seems that one source may be the nuclein of the food taken. Even if there is not time for digestion and conversion of the nuclein present in the food, a source for the uric acid may be found in the purine bases present there. Finally, some may come from the cells used up in the digestive glands. Again, he shows that the uric acid excreted in the urine is not an index of the amount produced in the body, for the liver, the muscles, and the kidneys have the power of destroying as well as producing it; other portions may be stored up and others are carried off in the fæces. Indeed all the purine compounds must be considered together, and it has been proved that a part of them is excreted independent of diet, while another part is due directly to the diet. The life history of all this group of bodies in health must be worked out, and then we may attack hopefully that of uric acid as one of them both in health and finally in disease. Smith Jerome thinks that all which we know certainly about the excretion of uric acid is that some is independent of the food, some i dependent on the quantity and quality of the food, and that the food which leads to an increase contains alloxuric nitrogen. A. Haig[32] records that he has examined his own urine for 2,990 days or more than eight years, and during this time he has passed 36,987 grains of uric acid and 29 times that amount of urea, or a daily average of 12 and 349 grains respectively. Of these 12 grains of uric acid excreted daily he calculates one is due to the amount previously in his body, together with some taken experimentally, while two are due to that taken contained in food, leaving nine or ten as the amount formed in the body daily. The use of tea and flesh food is, he holds, the cause of the introduction of uric acid in excess into the system and of the diseases which follow it.

W. Ringrose Gore[33] advances a theory that gout is not due to the presence of uric acid in the blood; its symptoms are caused by a toxin, which is produced by an intestinal bacillus acting on abnormal or dyspeptic excretions. The excess of uric acid is due to the action of this toxin on the liver. Weiss discovered that quinic acid, a constituent of many fruits, reduces the formation of uric acid in the body. This substance has been combined with piperazine and administered to gouty patients with marked success by Blumenthal, von Leyden[34] and others. The excretion of uric acid is reduced by 40 or 50 per cent. when sidonal (as the quinate of piperazine is called) is given in doses of 75 to 80 grains daily. The uric acid appears to be excreted as hippuric acid and is not stored up in the organism. The remedy does not produce any bad symptoms if given for several days together; the attacks pass off quickly and mildly, and the joints regain mobility at an earlier time than under other treatment. Similarly attacks of gravel are said to be relieved by its use. Colchicine is another substance which has been widely used of late, either alone or combined with the salicylate of methyl.

[23] Brit. Med. Journ., Nov. 10. [24] Practitioner, Dec. [25] Lancet, Nov. 3. [26] Med. Rec., Oct. 27. [27] Lancet, Dec. 8. [28] Med. Chron., Oct. [29] Med. Rec., Nov. 10. [30] Clin. Journ., Jan. 23. [31] Brit. Med. Journ., Sept. 29. [32] Loc. cit. [33] Brit. Med. Journ., Sept. 15. [34] Die Medicin Woche, Mar. 12, 1900.

